# The art of the clinical examination is still relevant in internal medicine at teaching hospitals in South Africa

**DOI:** 10.4102/jcmsa.v4i1.340

**Published:** 2026-02-10

**Authors:** Mogamat-Yazied Chothia

**Affiliations:** 1 Division of Nephrology, Department of Medicine, Faculty of Medicine and Health Sciences, Stellenbosch University, Cape Town, South Africa

**Keywords:** clinical medicine, clinical reasoning, training, internal medicine, specialisation

## Abstract

Clinical examination has long been central to diagnostic reasoning and to cultivating core professional attributes in internal medicine training; however, the relevance of clinical examination has been increasingly questioned in an era in which rapid access to specialised investigations is expanding. In the Eastern Metropole of Cape Town, the ‘decentralisation’ of specialised investigations, particularly radiological imaging and point-of-care ultrasound, has made these tools readily accessible at peripheral hospitals. While diagnostic efficiency has improved at peripheral centres, this improvement has occurred alongside a reduced emphasis on fundamental bedside skills among internal medicine registrars at the tertiary level. At our tertiary centre, the overburdened internal medicine admissions area leaves little time for detailed history taking or comprehensive physical examination. Consequently, investigations performed at referring centres often precede bedside assessment at our centre. This trend risks eroding the core competencies within internal medicine training, including diagnostic reasoning, observational proficiency and elements of the hidden curriculum such as communication, rapport-building and professionalism. In South Africa’s resource-constrained environment, in which clinicians often confront advanced disease and complex pathology, the clinical examination remains indispensable. Training programmes should reaffirm the vital role of clinical examination, ensuring that registrars in internal medicine maintain mastery of bedside assessment, rather than relying on special investigations. The art of clinical examination remains fundamental to good medical practice and should be actively preserved within teaching hospitals.

## Introduction

I am reminded of the great diagnostician, Dr Joseph Bell, a Scottish surgeon at the University of Edinburgh and the inspiration behind Sherlock Holmes, created by Sir Arthur Conan Doyle. Dr Bell possessed extraordinary observational skills, refining his diagnostic abilities daily by scrutinising seemingly insignificant details.^1^ His exceptional talent for discerning and connecting facts elevated his diagnostic and deductive reasoning skills to a renowned level. Another esteemed clinician, Sir William Osler, once said, ‘The art of medicine is in observation … but to educate the eye to see, the ear to hear and the fingers to feel takes time’. Unfortunately, these skills are increasingly being lost by physicians in contemporary medical practice.

## The effect of ‘decentralisation’ of special investigations on internal medicine training

Over the past decade in the Eastern Metropole area of Cape Town, South Africa, special investigations, particularly radiological imaging techniques, have become more readily accessible to physicians working in peripheral hospitals. I refer to this shift as the ‘decentralisation’ of specialised investigations, given that such tests were once limited to tertiary centres. The tertiary centre at which I work functions within a unique referral system, as the centre does not have an emergency department. Consequently, patients are referred from peripheral hospitals directly to specific departments that offer an acute or admissions service in which the diagnostic work-up, including several radiological investigations, has already been performed. Internal medicine registrars working in these emergency areas often lack the time to take thorough histories and perform comprehensive clinical examinations. This issue is exacerbated by the decentralisation of special investigations, significantly impacting the clinical skills of the registrars. Historically, clinical diagnosis relied primarily on a thorough history and physical examination. Internal medicine registrars dedicated more time to honing their clinical abilities by spending more time with patients, taking detailed histories and meticulously examining whether clinical signs were present. Clinical skills were calibrated *after* the results of special investigations became available, either confirming or refuting clinical findings. For instance, in a hypothetical patient with aortic regurgitation (AR) from suspected infective endocarditis (IE), the history would focus on risk factors for IE, while the clinical examination would highlight classic physical signs of AR and IE, such as wide pulse pressure, collapsing pulse, Duroziez sign, Corrigan sign, Quincke sign, splinter haemorrhages, Janeway lesions and Osler nodes, among others. Confirmation of these clinical findings by an echocardiogram would enhance the registrar’s clinical confidence. While confirmation provides a significant boost to the registrar’s confidence, refuting the clinical findings or revealing an alternative diagnosis offers an opportunity for the registrar to recalibrate their examination skills. On the other hand, this process has been accelerated by using point-of-care ultrasound (POCUS).

In recent years, the decentralisation of special investigations, such as computed tomography (CT) and echocardiography, may have significantly impacted the clinical skills of internal medicine registrars at our training centre. Additionally, POCUS is gaining popularity among younger colleagues and may be surpassing traditional physical examinations and replacing the stethoscope. The enhanced diagnostic accuracy of POCUS for common conditions such as pleural effusion, pulmonary oedema, pulmonary embolism, pneumonia, congestive heart failure, ascites and lower limb deep vein thrombosis has been clearly demonstrated.^2^ Furthermore, as a result of the proven ability of POCUS to expedite diagnosis, lower hospital costs and possibly reduce the length of hospital stay, POCUS presents an appealing alternative. However, POCUS is not included in the formal internal medicine training programme at our centre. Consequently, our teaching hospital has shifted its focus from diagnostic dilemmas to therapeutic interventions. With many patients now receiving initial diagnoses at peripheral centres, referrals for ongoing therapeutic care, exclusive to tertiary centres, have increased. As a result, the time spent by internal medicine registrars around the bedside has diminished, with more time allocated to computer tasks such as requesting, interpreting or following up on radiological and laboratory reports.

This reliance on special investigations has stolen the time that our clinicians spend with patients. This shift could significantly affect both the patient–doctor relationship and the clinical reasoning. Mastery of skills such as gathering information through history-taking and physical examinations should precede the interpretation of CT scans or laboratory results^3^; however, this activity is frequently performed in reverse as patients are now referred to tertiary centres with completed special investigations and diagnoses. A prospective study assessed the diagnostic capabilities of a senior resident who relied solely on history, physical examination and basic ancillary tests, excluding POCUS, to diagnose adult patients admitted from the emergency department to the internal medicine ward.^4^ Correct diagnoses were achieved in over 80% of admissions, with an average time of 40 min spent per patient. The most effective methods for accurate diagnosis were found to be history alone (20%) and combined history with physical examination (40%).

Another key consideration is the assessment of clinical competence, a component of the Fellowship of the College of Physicians’ internal medicine exit examination. This assessment evaluates candidates’ ability to take a history, perform a physical examination and generate a diagnosis or differential diagnoses based solely on these findings. Overreliance on special investigations during training may adversely affect registrars’ performance in this examination. Therefore, during their training, internal medicine registrars should assess patients at the bedside, despite existing investigations, to minimise cognitive biases like anchoring and confirmation bias, thereby preserving essential clinical skills in an investigation-driven environment.

Furthermore, interacting with patients fosters the acquisition of essential skills beyond medical knowledge, known as the hidden curriculum. These encompass developing communication skills, establishing rapport and trust and internalising professional values.^3,5^ Despite the importance of these skills, clinicians in internal medicine may spend as little as 12% of their time directly engaging with patients, with 40% of their time consumed by computer-related tasks.^6^

## Conclusion

South African healthcare providers face significant demands as a result of the prevalence of complex pathologies that often manifest late in their progression. Clinicians are thus expected to possess a wide range of skills, frequently tackling challenges beyond their specific expertise. Clinicians, including those in the private sector constrained by medical aid limitations on diagnostic tests, operate in resource-limited environments. While clinical settings prioritise efficient patient service and throughput, this narrative requires a shift in focus. Registrars in internal medicine should remember that their primary goal is to learn and master their chosen speciality. Investigations are an essential part of the diagnostic process, and the emphasis should be on using them responsibly to complement clinical findings and facilitate timely diagnosis, ensuring optimal patient care; however, the clinical examination has always been and will continue to be at the heart of the practice of internal medicine.
